# The distributional effects of power outages on regional economies: evidence from dynamic panel data and machine learning models

**DOI:** 10.1007/s00168-026-01500-y

**Published:** 2026-05-02

**Authors:** Sanjay Singh, Zachary Keeler, Bradley Ewing

**Affiliations:** 1 Harrisburg University of Science and Technology, Harrisburg, United States; 2 Texas Tech University, Lubbock, United States

## Abstract

We examine the linkages between power reliability, economic growth, and income inequality in the United States. Specifically, we use the two-step System Generalized Method of Moments (GMM) estimator to assess the impact of power interruptions on state-level GDP and the Gini Index. Our findings reveal that a 1 percent increase in power interruptions, measured in terms of duration (SAIDI) and frequency (SAIFI), is associated with a 0.07 to 3.7 percent decrease in real GDP and a modest increase in income inequality of approximately 0.17 to 0.20 percent relative to the mean Gini Index. Moreover, the marginal effects of power interruptions are substantial, with frequent outages resulting in GDP losses exceeding $2 trillion in the long run. We also use machine learning models to support the predictive relevance of the power reliability metrics. Overall, the results highlight the significant role that both the frequency and duration of power interruptions play in shaping regional economic performance and the importance of improving power reliability to foster economic stability and equity.

## Introduction

Energy is essential to economic growth and development in our modern society. However, power interruptions (i.e., loss of electric power to homes and businesses) are a recurring challenge faced by communities and economies around the globe.^1^ These interruptions in electricity supply disrupt daily life and significantly affect local businesses and individual outcomes (Barnes et al. 2011; Chen et al. 2022; Diboma & Tatietse 2013; Vennemo^2022^ ). Given that energy is fundamental to all sectors and underpins all economic activity (Atems & Hotaling 2018), understanding how disruptions to the electric power system are associated with regional economic growth is of considerable policy importance.

Numerous studies have explored the relationship between electricity and economic outcomes, particularly at the macro level. Much of the literature has focused on Granger causality between electricity consumption/generation and growth (e.g., Acaravci & Ozturk 2012; Apergis & Payne 2010; Kraft & Kraft 1978; Pao & Fu 2013). While the findings are somewhat mixed, previous studies consistently suggest linkages between energy and economic performance.

The reliability of electricity also appears to play a role in the success of local businesses and economies. For example, power outages have been shown to have a negative relationship with firm sales (Cole et al. 2018) and productivity (Fisher-Vanden et al. 2015; Moyo, 2012). Additionally, several studies have examined the relationship between power reliability and gross domestic product (GDP). For example, outages have been shown to be associated with lower GDP growth rates (Abeberese 2017; Andersen & Dalgaard 2013; Atems & Hotaling 2018). However, this body of research mainly includes case studies conducted outside the United States (U.S.), which results in the U.S. context being understudied, despite its decentralized energy governance and regional variations in power reliability.

The consequences of power interruptions are also evident at the individual and household levels. Power interruptions can impact essential services like lighting, heating or cooling, refrigeration, and communication (Nduhuura et al. 2021; Stock et al. 2023). Thus, households face additional challenges during interruptions, likely causing a reduction in disposable income, potentially negatively affecting consumption patterns and overall living standards. Moreover, as the distribution of power outages is not uniform across the U.S., the effects of outages may differ across communities and regions and disproportionately impact lower-income groups (Dugan et al. 2023; Lee et al. 2022).

Given the importance of energy and its reliability to economic and individual outcomes, we use the System Generalized Method of Moments (System GMM) Arellano-Bond estimator to assess how power interruptions are associated with state-level GDP and income inequality (Gini Index) in the U.S. Specifically, we use various measures of power reliability from the U.S. Energy Information Administration related to the duration and frequency of outages from 2013–2022 to explore the linkages between interruptions to the power system and state-level outcomes over the same period.

Our results indicate that power interruptions are negatively associated with economic growth and positively associated with the Gini Index (representing greater income inequality). Specifically, we find that a 1% increase in power interruption, measured by duration or frequency, is associated with a 0.07%–3.7% decrease in GDP and an increase in income inequality of 0.17%–0.20% relative to the mean Gini Index. Additionally, there appear to be stronger effects when interruptions due to major event days (e.g., hurricanes or other disasters) are excluded from the analysis. In other words, the estimates suggest significant losses to the economy, especially when the outages are relatively more avoidable through routine maintenance, operational procedures, etc.

In addition to exploring this relatively understudied topic in the U.S. by using the System GMM Arellano-Bond estimator (which, to our knowledge, its application to the power reliability–inequality–growth nexus in the U.S. context is novel), we also employ three different machine learning-based robustness checks. Overall, these predictive validation models provide strong complementary support for our main results.

From a theoretical perspective, our study aligns with frameworks in economic resilience and institutional economics. Economic resilience theory suggests that regions’ ability to withstand and recover from shocks, such as infrastructure failure, has significant implications for long-run growth and inequality (Rose 2007). Power interruptions can be viewed as negative infrastructure shocks with regionally varied impacts depending on state capacity, grid investments, and institutional quality (Herrera-Catalán, Chasco, & Royuela, 2025). Institutional economics also highlights how discrepancies in public infrastructure and regulatory capacity affect development outcomes (North, 1990; Calderón & Chong 2004). Our focus on power reliability expands this literature by quantifying how routine service quality—rather than infrequent blackouts—impacts economic performance and income distribution. By understanding these impacts, stakeholders can work toward improving electricity access and reliability to foster better regional growth.

The remainder of the paper proceeds as follows. Section 2 provides a review of related literature, Sect. 3 discusses the data, Sect. 4 describes the empirical method, Sect. 5 presents the results, and we conclude with final remarks in Sect. 6.

## Previous literature

Several studies have explored the relationship between electricity consumption and growth (Acaravci & Ozturk 2012; Apergis & Payne 2010; Kraft & Kraft 1978; Pao & Fu 2013). Other research suggests that the impact of electricity generation on growth may differ from that of consumption. For example, Atems & Hotaling (2018) estimate the effect of electricity generation on economic growth in 174 countries using the System GMM estimator. Their results indicate a strong positive and statistically significant relationship between electricity generation (both renewable and non-renewable) and GDP per capita growth.^2^

While there is certainly evidence of a link between energy and economic outcomes, the reliability of electricity also appears to be an important consideration. For example, Cole et al. (2018) use firm-level data for 14 African countries and find that power outages have a negative relationship with firm sales, especially for those businesses that lack a substitute for the electricity (i.e., power generators). Moyo (2012) finds that power outages negatively affect productivity, particularly for small firms in Nigeria. Similarly, Fisher–Vanden et al. (2015) suggest that power shortages in China result in low firm productivity.

Abeberese (2017) examines the productivity and performance of manufacturing firms in India and finds a negative linkage between power outages and firm productivity, resulting in a lower GDP growth rate. Andersen & Dalgaard (2013) also find substantial growth drag due to poor power infrastructure when employing lightning density as an instrument for power outages in Sub-Saharan African countries. Linkages between electricity loss and economic growth are also found in other studies (Atems & Hotaling 2018).

One of the major challenges the electricity sector faces is weather-induced power outages. For example, Bhattacharyya, et al. (2021) look at the effects of severe weather-induced power outages in the U.S. and find an estimated $11.6 billion loss in GDP due to 1% inoperability in the utility sector. The authors highlight the importance of a reliable electricity supply for maintaining economic stability. In regional-specific studies, Bhattacharyya & Hastak (2022) estimate the expected loss of the 2021 Texas winter storm to be approximately $664 million. Rose et al. (1997) use a simulation to assess the regional economic impacts of electricity lifeline disruptions caused by a catastrophic earthquake near Memphis, Tennessee, and find that potential production loss over the recovery period could amount to as much as 7 percent of gross regional product. Other research has estimated the costs of large electricity outages, including case studies of hurricanes (Henry & Ramirez-Marquez 2016; Wanik 2018; Xiao et al. 2021). Power outages are not only an issue in the U.S. but also in other parts of the world. For example, Chen et al. (2022) and Diboma & Tatietse (2013) find large business interruption costs due to power outages in China and Cameroon, respectively.

Power interruptions also have implications at the individual and household levels. Several studies have used contingent valuation surveys to estimate the value that households place on having reliable power (Aweke & Navrud 2022; Hensher 2014; Kim 2015; Lehmann et al. 2022; Vennemo^2022^; Stock et al. 2023). Overall, these papers suggest that households are willing to pay a premium to avoid power outages.

Previous research also suggests that the effects of power outages may differ across communities. For example, Dugan et al. (2023) find that there may be various socioeconomic and demographic characteristics, such as income levels, that can affect access to proper resources and levels of preparedness. Additionally, Lee et al. (2022) find a disparity in the extent and duration of power outages experienced by low-income and minority groups in Harris County during the February 2021 Texas winter storm. Singh et al. (2024) evaluate the policy implications of renewable portfolio standards (RPS) for grid reliability in the U.S. using an instrumental variable quantile framework. They find that RPS policies can have heterogeneous effects on reliability, underscoring the need to consider both policy design and regional system characteristics when assessing resilience. Outside of the U.S., Aweke & Navrud (2022) look at both urban and rural areas of Northern Ethiopia and find that households’ willingness to pay to avoid blackouts increases with income. The reliability of electricity has also been found to have significant positive effects on the socioeconomic empowerment of women in India, but the effects differ between location, living standards, and education (Sedai et al. 2022). Rural electrification has also been found to facilitate structural transformation of village economies in Ethiopia and slowed out-migration from rural areas (Fried & Lagakos 2021). Thus, there may be differences between areas, including income levels that exacerbate the impacts of outages.

## Data

### Power reliability data

The power reliability data utilized in this study were sourced from the U.S. Energy Information Administration (EIA), extracted from their Annual Electric Power Industry Report (Form EIA-861). This survey collects data from over 3,600 distribution utilities and power marketers of electricity from 2013 to 2022. Electric distribution utilities collect and self-report data when there is a power outage (i.e., reliability). Specifically, utilities report the system average interruption duration index (SAIDI), the system average interruption frequency index (SAIFI), and the customer average interruption duration index (CAIDI).^3^ Both SAIDI (number of minutes of interruption the average customer experiences) and SAIFI (average number of times a customer experiences an outage) are the yearly summed values of all non-momentary outages.^4^ CAIDI can be derived from the SAIDI and SAIFI values, and measures the average duration of an interruption.^5^ These metrics are reported by utilities using the standardized IEEE 1366 methodology, and median imputation combined with consistent filtering ensures comparability across U.S. states over the 2013–2022 period.

Under EIA Form 861, utilities report SAIDI, SAIFI, and CAIDI as standardized reliability outcome measures following IEEE Standard 1366. These metrics do not explicitly model the causes of outages, such as weather conditions, temperature extremes, vegetation, or economic activity, but instead capture the realized performance of the electric distribution system. As a result, the reported reliability outcomes reflect the combined effects of grid quality, operational practices, economic structure, and exogenous shocks, including weather-related events.

All three measures of power reliability are reported by the utilities ‘with’ and ‘without’ major event days (MED).^6^ ‘With MED’ (w/ MED) includes all non-momentary outages, regardless of why the outage occurred. ‘Without MED’ (w/o MED) removes some of the outages, specifically those often caused by bigger and more unavoidable events (e.g., large storms). In other words, ‘without MED’ provides a better picture of what might be referred to as normal operations.

The Major Event Day (MED) classification in IEEE 1366, as implemented in EIA-861 reporting, identifies days with statistically extreme outage activity that are typically associated with severe weather or large-scale disturbances. By analyzing reliability metrics both with and without MED, we distinguish routine, operational grid reliability from outages driven by extreme exogenous events. This approach provides an indirect but systematic way to account for weather exposure while maintaining consistency and comparability across states.

**Fig. 1:**
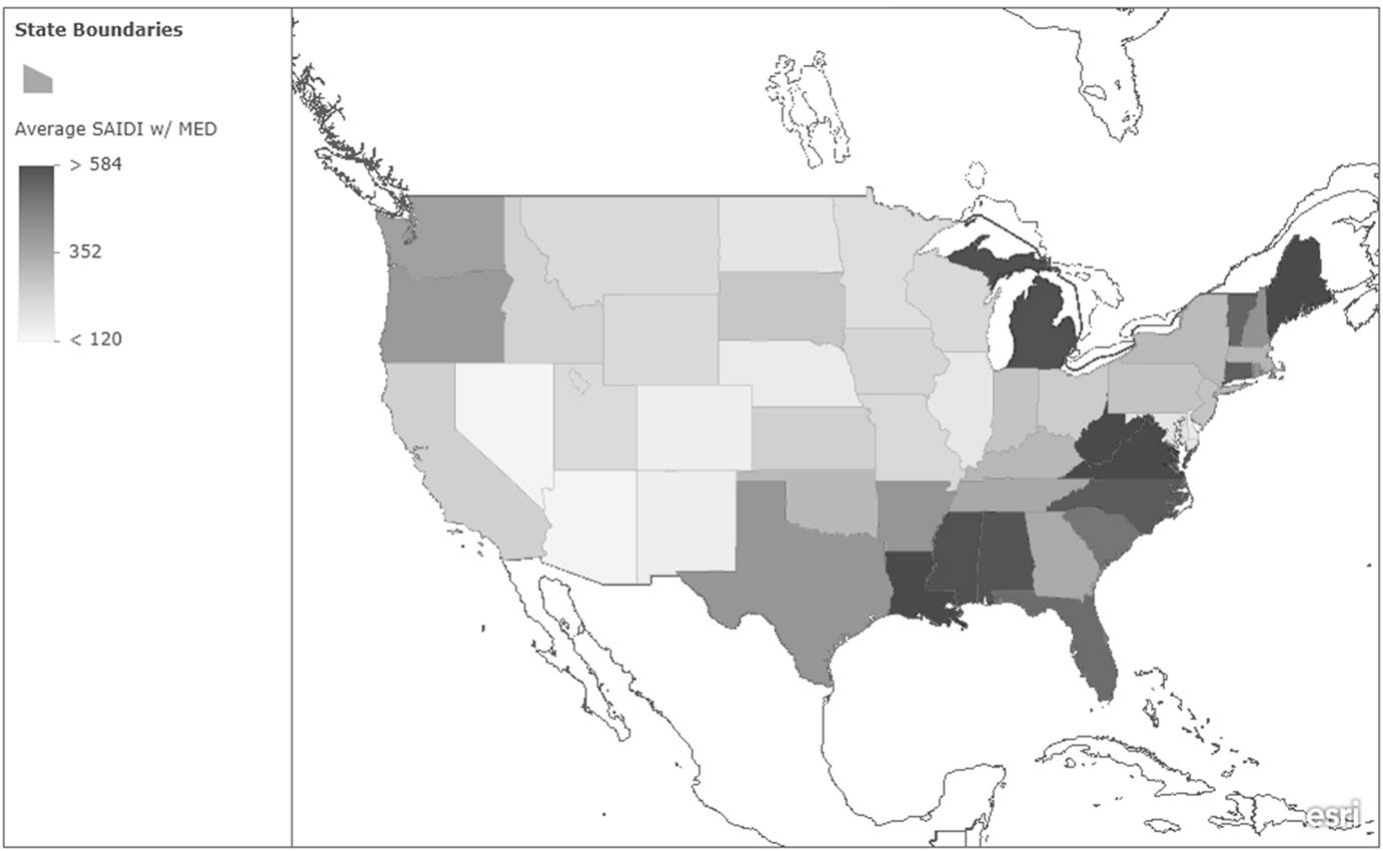
Average SAIDI with MED (2013–2022). *Notes*: This figure shows the spatial variation in the state-level average SAIDI with MED. Higher values indicate less reliable power (i.e., values indicate the number of minutes of interruption the average customer experiences per year)

**Fig. 2:**
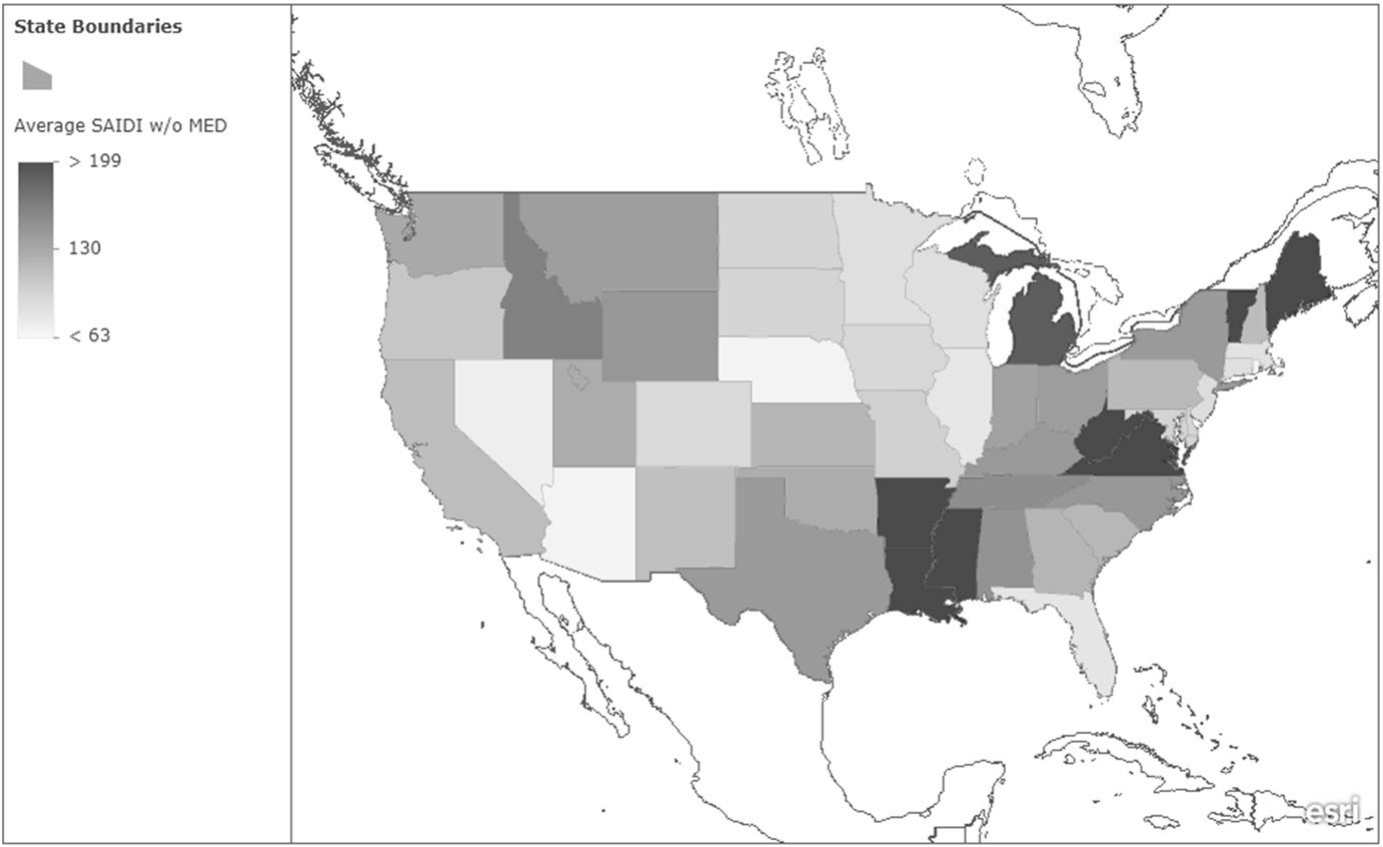
Average SAIDI without MED (2013–2022). *Notes*: This figure shows the spatial variation in the state-level average SAIDI with MED. Higher values indicate less reliable power (i.e., values indicate the number of minutes of interruption the average customer experiences per year)

The average state-level SAIDI with MED is presented in Fig. 1. As the figure shows, there is tremendous spatial variation in power reliability. The figure shows that SAIDI with MED varied from 93 min (Arizona) to over 1,366 min (Louisiana) of interruption. The southern states, including Florida, appear to have relatively longer power outages, on average. However, Fig. 1 displays power reliability for all types of outages, including those that may make the South more susceptible (e.g., hurricanes). Figure 2 presents SAIDI without MED, which provides a better picture of how reliable power is on a more consistent basis. As the figure shows, many of the states that had less reliable power when MED was included tend to have relatively lower SAIDI values when it is excluded. The differences between these figures provide further evidence that it is important to examine power reliability with and without MED separately.

### Macroeconomic, Demographic, and Structural Covariates

Macroeconomic and demographic covariates are sourced from various publicly available resources to account for structural heterogeneity among U.S. states. Real Gross Domestic Product (GDP) and GDP per capita, both provided by the Bureau of Economic Analysis (BEA), serve as proxies for aggregate and per capita economic output, respectively. These indicators reflect the scale and intensity of economic activity that may be influenced by power reliability shocks.

Income inequality is measured using the Gini Index from the American Community Survey (ACS), which quantifies deviations from a perfectly equal income distribution. The index ranges from 0 (perfect equality) to 1 (perfect inequality) and is widely used to assess the distributional consequences of macroeconomic and infrastructural changes (Group 2020). This approach is consistent with prior evidence that infrastructure volume and quality significantly influence income distribution across countries (Calderón & Chong 2004).

Labor market conditions are controlled for using the state unemployment rate from the Bureau of Labor Statistics (BLS). Higher unemployment is associated with weaker household demand, fiscal stress, and increased sensitivity to infrastructure failures (Blanchard & Katz 1997; Gali 2011; Carroll et al., 2019). Total state population (log-transformed) is included to capture scale effects in infrastructure demand and public service delivery (Bloom, Canning, Fink, & Finlay, 2007). The GDP regressions also include the manufacturing share, labor force participation rate, broadband access rate, urbanization rate, electricity price, and capital outlay per capita as control variables while the Gini Index regressions retain electricity price and capital outlay per capita to account for variation in infrastructure costs and public investment across states.^7^

Overall, this covariate set supports a robust identification strategy by accounting for key socioeconomic and structural factors that may jointly influence power reliability and economic performance. Detailed descriptions, measurement units, and data sources for all variables, including justification for their inclusion, are provided in Appendix Table A.

Before applying the Arellano–Bond estimator, we converted the power reliability measures SAIDI and CAIDI, originally provided in minutes, into hours for consistency in analysis and interpretation. However, SAIFI, which is measured in terms of the number of interruptions, has been retained in its original form. Subsequently, we transformed the data into log form, except for the unemployment rate and Gini Index, to facilitate a more accurate estimation of elasticities and relationships between the variables. This transformation was implemented to mitigate the dimensional effect and to meet key model assumptions, including no second-order autocorrelation in the differenced residuals, validity of instruments as confirmed by the Hansen J-test, correct model specification, stationarity of variables, and homoscedastic residuals. Additionally, by transforming the data into logarithmic form, we address issues related to heteroscedasticity and nonlinearity, enabling more accurate and reliable estimation results.

Table 1 reports summary statistics for the key variables used in the analysis.^8^ On average, SAIDI w/MED is approximately 1.66 h, with a standard deviation of 0.63, while SAIDI w/o MED averages 1.10 h. As expected, outages tend to last longer when major events are included. Similarly, the average CAIDI w/ MED is 1.39 h, compared to 1.03 h w/o MED. The average SAIFI w/ MED is 0.88 interruptions and 0.74 w/o MED. These relatively low means reflect a moderate level of reliability across U.S. states during the study period, though the variation remains notable.

**Table 1:** Descriptive statistics of key variables

Variable	Mean	Std. Dev	Min	Max
SAIDI w/ MED	1.655	0.633	0	4.593
SAIDI w/o MED	1.101	0.311	0	2.309
CAIDI w/ MED	1.391	0.436	0	3.406
CAIDI w/o MED	1.034	0.178	0	1.753
SAIFI w/ MED	0.876	0.221	0	1.808
SAIFI w/o MED	0.742	0.182	0	1.482
Real GDP	12.325	1.048	10.358	14.968
Gini Index	0.465	0.019	0.41	0.52

All values are presented in their logarithmic form, except the Gini Index. Also note that 'w/' denotes 'with,' and 'w/o' denotes 'without,' as used in the table to indicate the inclusion or exclusion of specific factors

Turning to the outcome variables, Real GDP has a mean of 12.33 (in log thousands of chained 2017 dollars), and the Gini Index averages 0.465, with a relatively narrow standard deviation of 0.019, consistent with prior findings on persistent state-level inequality patterns in the U.S.

## Econometric approach

To assess how power reliability is associated with GDP and the Gini Index across the 50 U.S. states from 2013—2022, we use the System Generalized Method of Moments (GMM) method proposed by Arellano & Bover (1995) and Blundell & Bond (1998). The choice of System GMM is motivated by the dynamic nature of the dependent variables and the presence of unobserved state-specific effects, which render standard fixed-effects estimators biased in panels with a limited time dimension due to the well-known Nickell (1981) bias. Random effects specifications were considered but rejected because unobserved state-specific factors are likely correlated with infrastructure quality and power reliability measures. While alternative estimators such as the Augmented Mean Group (AMG)^9^ approach have been proposed in the literature to address dynamic bias under cross sectional dependence, our primary identification strategy relies on System GMM because it explicitly corrects for endogeneity arising from lagged dependent variables and potential simultaneity between power reliability and economic outcomes using internal instruments. Although income inequality evolves more slowly than GDP, it exhibits strong persistence due to institutional and labor market rigidities; a dynamic specification therefore mitigates omitted variable bias and captures gradual adjustment processes in inequality outcomes.

To further ensure that the results are not driven by the assumptions underlying dynamic panel GMM estimation or by instrument choice, we complement the System GMM analysis with machine learning-based models, including Ridge regression, Random Forest, and XGBoost. Unlike fixed-effects estimators with lagged dependent variables, these machine learning models are not subject to Nickell bias and do not rely on instrumental variables. The consistency of findings across GMM and nonparametric machine learning frameworks provides an additional robustness check and strengthens confidence in the substantive relationship between power reliability, economic growth, and income inequality.

This estimator is designed for panels with many cross sectional units and relatively few time periods. It also corrects for dynamic panel bias, potential endogeneity of regressors, and autocorrelation in the idiosyncratic errors. We use System GMM over Difference GMM because it improves estimation efficiency by utilizing additional moment conditions from both the level and differenced equations, creating more robust instruments, particularly for persistent variables (Nickel1, 1981). This approach also reduces finite sample bias common in Difference GMM, provides greater flexibility in instrument selection, and yields more reliable estimates (Arellano & Bond 1991; Blundell & Bond 1998; Bond 2002). To avoid instrument proliferation, we use the collapse option in Stata as recommended by Roodman (2009).

In a dynamic panel data estimation, a simple model with one lag of the dependent variable as a regressor with a vector of other regressors is:

1 $$y_{it} = \alpha y_{{\left\{ {i,t - 1} \right\}}} + \beta x_{{\left\{ {i,t} \right\}}} + \Upsilon z_{{\left\{ {i,t} \right\}}} + u_{i} + \varepsilon_{{\left\{ {i,t} \right\}}} ; \forall i = 1, \ldots ,N, T = 1, \ldots ,T$$

Taking the first difference of Eq. (1) to eliminate individual effects gives:

2 $$\Delta y_{it} = \alpha \Delta y_{{\left\{ {i,t - 1} \right\}}} + \beta \Delta x_{{\left\{ {i,t} \right\}}} + \Upsilon \Delta z_{{\left\{ {i,t} \right\}}} + \Delta \varepsilon_{{\left\{ {i,t} \right\}}}$$

where ${y}_{it}$ is the dependent variable (either the natural log of GDP or the Gini Index) in state *i* and time *t*; ${x}_{it}$ denotes the log of the various power reliability measures (the endogenous variables include log- transformed GDP and untransformed Gini Index); and ${z}_{it}$ is a vector of control variables (e.g., population, unemployment, and others). To address potential endogeneity and ensure consistent parameter estimates, GMM-style instruments are created using lagged values of endogenous variables, allowing the model to control for endogeneity by leveraging past information. This has advantages over other types of instruments, such as IV-style instruments, which are often external or predetermined variables that are assumed to be uncorrelated with the error term. In System GMM, these are applied in the level equation, where they help correct for potential biases in the estimation process. Combining these instruments enhances model efficiency and robustness.

Additionally, the potential for bidirectional causality between electricity reliability and economic outcomes—for instance, less reliable power may diminish GDP, while reduced economic activity may also restrict investments in grid infrastructure—requires the use of internal instruments based on lagged levels and differences. This approach reduces simultaneity bias and strengthens the causal interpretation of our estimates.

The ${u}_{i}$ are the panel-level effects or the fixed effects that may be correlated with the covariates, and ${\upepsilon}_{it}$ is the independent and identically distributed error term $(\upepsilon \sim N\left(0,{\sigma}_{\upepsilon }^{2}\right);\forall i and t.$ The coefficients α are expected to be between 0 and 1 and β are the estimated coefficients. It is also assumed that error terms are serially uncorrelated. Each model uses robust standard errors clustered by the states.^10^

In the GDP models, the lagged dependent variable (log GDP per capita) is treated as endogenous, adhering to standard practice in dynamic panel modeling where persistence and feedback effects are anticipated (Blundell & Bond 1998). Core regressors—such as power reliability indicators (SAIDI, SAIFI, and CAIDI) and their interactions with GDP per capita—are also considered endogenous due to the potential for bidirectional causality, which allows economic performance to influence infrastructure reliability and vice versa. Additionally, variables like manufacturing output and its interaction terms derive endogeneity from their components. Labor market and demographic variables—including the unemployment rate, population size, labor force participation, and broadband access—are classified as predetermined, as past economic conditions affect them but are unlikely to react immediately to shocks in the dependent variable. Structural variables—such as electricity access, urbanization rate, and capital outlay per capita—are regarded as exogenous, since they change gradually over time and are less vulnerable to short-term fluctuations in GDP (Nickell 1981).

The income inequality models follow a comparable specification. The Gini Index and power interruption indicators are modeled as endogenous, while their labor market controls (e.g., unemployment rate) are treated as predetermined variables that may be influenced by past, but not current, realizations of inequality. Structural controls such as capital outlay per capita and electricity access are again treated as exogenous. The classification of variables is based on both theoretical expectations and empirical diagnostics (Roodman 2009).^11^

It is important to note that in our analysis, we employed different sets of instruments for the power interruption variables to ensure the validity and relevance of the instruments for each specific context. Given that the power interruption variables capture distinct dimensions of power reliability, such as frequency and duration, it was necessary to tailor the instruments to reflect these differences. The robustness of our results, confirmed by the Hansen J-test presented in our results tables, supports the use of these instruments. All models are estimated using robust standard errors and are accompanied by post-estimation diagnostics, including the Arellano-Bond tests for first- and second-order serial correlation and tests for overidentifying restrictions.

## Results

### Power reliability and state-level GDP

To examine how power reliability affects state-level economic performance, we estimate Eq. (1) using System GMM, successively replacing the power reliability metric (SAIDI, SAIFI, and CAIDI) in each specification, both with and without the inclusion of major event days (MED). Because both state-level GDP and the power reliability measures are log-transformed, the estimated coefficients in this section are interpreted as elasticities. The results, reported in Table 2, reveal a clear and consistent pattern: higher values for power interruption variables, indicating less reliable power, are associated with statistically significant reductions in state-level GDP. This finding holds across all specifications and both estimation techniques (one-step and two-step GMM), reinforcing the robustness of the observed effects.

**Table 2:** Power reliability and state-level GDP (2013—2022)

Dependent Variable: GDP						
Variable	SAIDI w/ MED	SAIFI w/ MED	CAIDI w/ MED	SAIDI w/o MED	SAIFI w/o MED	CAIDI w/o MED
*One-Step System GMM*						
Lagged GDP	0.934*** (0.135)	0.318*** (0.080)	0.891*** (0.118)	0.804*** (0.058)	0.287*** (0.087)	0.819*** (0.063)
Power Interruption	− 0.073** (0.036)	− 3.193*** (0.345)	− 0.832** (0.038)	− 0.110*** (0.040)	− 3.677*** (0.446)	− 0.154** (0.066)
Unemployment Rate	− 0.528* (0.290)	0.279 (0.194)	− 0.430* (0.246)	− 0.146 (0.156)	0.210 (0.161)	− 0.094 (0.140)
Population	0.061 (0.119)	0.678*** (0.076)	0.089 (0.100)	0.180*** (0.050)	0.701*** (0.090)	0.168*** (0.051)
Manufacturing Share	0.003 (0.024)	0.009 (0.022)	0.011 (0.023)	0.033** (0.015)	0.027 (0.020)	0.030* (0.016)
LF Participation Rate	− 0.521* (0.290)	0.280 (0.194)	− 0.423* (0.246)	− 0.137 (0.155)	0.212 (0.160)	− 0.085 (0.139)
Broadband Access Rate	0.002*** (0.0001)	0.001 (0.001)	0.001** (0.0005)	0.00004 (0.0001)	− − 0.0002 (0.0002)	0.00006 (0.0001)
Power Interruption*GDP per Capita	0.002 (0.004)	0.783*** (0.082)	0.003 (0.003)	0.006*** (0.002)	0.892*** (0.110)	0.008** (0.003)
AR (1) test	0.003	0.000	0.001	0.000	0.001	0.000
AR (2) test	0.240	0.393	0.166	0.132	0.534	0.057
Hansen J-test	0.053	0.108	0.112	0.175	0.165	0.156
Difference-in-Hansen test	0.129	0.539	0.110	0.991	0.693	0.985
Controls	Yes	Yes	Yes	Yes	Yes	Yes
Instruments	28	28	28	52	28	52
Dependent Variable: GDP						
*Two-Step System GMM*						
Lagged GDP	0.959***(0.242)	0.253*** (0.093)	0.827*** (0.072)	0.800*** (0.062)	0.172** (0.085)	0.816*** (0.063)
Power Interruption	− 0.079** (0.035)	− 3.517*** (0.449)	− 0.023** (0.010)	− 0.110** (0.043)	− 4.167*** (0.469)	− 0.150** (0.072)
Unemployment Rate	− 0.371 (0.410)	0.166 (0.181)	− 0.208 (0.152)	− 0.158 (0.163)	0.178 (0.140)	− 0.097 (0.158)
Population	0.021 (0.198)	0.748*** (0.090)	0.166*** (0.060)	0.181*** (0.053)	0.812*** (0.088)	0.170*** (0.051)
Manufacturing Share	0.003 (0.044)	0.007 (0.020)	0.019 (0.018)	0.034**(0.015)	0.022 (0.016)	0.031* (0.017)
LF Participation Rate	− 0.366 (0.410)	0.168 (0.179)	− 0.199 (0.151)	− 0.149 (0.162)	0.180 (0.140)	− 0.088 (0.157)
Broadband Access Rate	0.001 (0.0009)	0.0002 (0.0002)	0.00009 (0.0002)	0.00003 (0.0001)	− 0.00008 (0.0002)	0.00006 (0.0001)
Power Interruption*GDP per Capita	0.003 (0.004)	0.851*** (0.106)	0.001 (0.001)	0.006** (0.002)	1.006*** (0.109)	0.008** (0.004)
AR (1) test	0.001	0.006	0.000	0.000	0.002	0.000
AR (2) test	0.961	0.561	0.014	0.170	0.367	0.072
Hansen J-test	0.053	0.108	0.052	0.175	0.165	0.156
Difference-in-Hansen test	0.129	0.539	0.924	0.991	0.693	0.985
Controls	Yes	Yes	Yes	Yes	Yes	Yes
Instruments	28	28	44	52	36	52
No. of Groups	50	50	50	50	50	50
Observations	450	450	450	450	450	450

^*^, ** and *** denote significance at the 10%-, 5%- and 1%-level. Each model regresses the log GDP on the log of lagged GDP and the log of the power reliability variable by replacing each one while controlling for the urbanization rate, electricity price, and capital expenditure per capita in Eq. (1). Robust standard errors clustered at the state-level are in parentheses. LF refers to the labor force

Among the three power reliability metrics, SAIFI (which measures power outage frequency) consistently exhibits the most pronounced negative association with GDP. For example, in the one-step GMM model, a one-unit increase in SAIFI (w/ MED) is associated with an estimated 3.19% decline in state-level GDP, while the two-step model indicates an even larger impact of approximately 3.52%, both statistically significant at the 1% level. Interpreted differently, a 1% increase in SAIFI (w/ MED) corresponds to a roughly 3.2–3.5% reduction in GDP, underscoring the outsized economic consequences of frequent outages. Using the mean GDP, these results suggest that each additional outage event is associated with a $7.9 billion reduction in state-level GDP.

This power interruption frequency effect is notably stronger than those observed for SAIDI (average outage duration) or CAIDI (average restoration time). For example, focusing on the two-step models, a 1% increase in SAIDI (w/ MED) leads to a GDP reduction of about 0.079%, while CAIDI (w/ MED) results in an approximate 0.023% decline. This suggests that, on average, for each additional hour of interruption, state-level GDP declines $9.36 billion. Perhaps these differences suggest that the frequency of interruptions imposes a more severe disruption on economic activity than the length or restoration time of individual outages. One possible explanation is that frequent outages impose repeated operational “start-up” costs, such as restarting equipment, reinitializing systems, and rescheduling workflows. This mechanism is consistent with the concept of dynamic economic resilience (Rose A., 2007) and with findings from organizational resilience research, which highlight that inadequate preparedness and continuity practices can magnify the costs of repeated disruptions (Ali et al. 2023).

We also find that the estimated effects are larger when major event days are excluded. For example, the results suggest that a 1% increase in SAIFI (w/o MED) is associated with a 4.17% decrease in GDP, which is considerably higher than the SAIFI impact with MED (3.52%). We also find larger effects for SAIDI and CAIDI w/o MED, compared to when major events are included. While it appears that economies tend to fare better when power providers are better able to limit and respond to interruptions regardless of why they occur, states especially benefit when their providers are better able to handle outages that may be deemed more avoidable.

To further investigate the differential economic effects of power interruptions, we include an interaction term between the power interruption variable and state-level GDP per capita. The motivation behind this is to test whether economically stronger states are more resilient to the negative consequences of unreliable power supply. The interaction term captures the extent to which a state’s income level moderates the economic impact of outages. The results show that the interaction term is positive and statistically significant across most of the power reliability metrics, indicating that the GDP-depressing effect of interruptions is weaker in states with higher GDP per capita.

This finding has important economic and policy implications. States with higher per capita income likely have better-developed infrastructure, more diversified economic bases, and greater fiscal capacity to buffer against supply shocks. As such, they are better positioned to absorb the negative effects of unreliable power without experiencing the same degree of economic disruption. In contrast, lower-income states are more vulnerable—lacking the infrastructure, capital redundancy, or administrative capacity to adapt. For example, in the case of SAIFI (w/MED), the interaction coefficient of approximately 0.783 in the one-step model and 0.851 in the two-step model implies that as GDP per capita rises, the GDP loss associated with increased outage frequency is significantly mitigated.

The positive coefficients of the interaction terms provide strong evidence that economic resilience, proxied by GDP per capita, plays a critical role in reducing the vulnerability of states to infrastructure-related disruptions. In essence, states with more economic resources are better equipped to shield themselves from the adverse consequences of power interruptions, while poorer states bear a disproportionate burden. This suggests that infrastructure unreliability may exacerbate regional economic disparities over time. For example, during 2013–2022, California—the state with the highest average GDP per capita—experienced an average SAIFI of 2.09, compared to 3.26 in Vermont, the state with the lowest GDP per capita. This difference highlights how residents in lower-income states face more frequent interruptions, magnifying the economic challenges posed by unreliable infrastructure.

Overall, the results indicate that power interruptions have a clear and negative impact on state-level GDP, and that the extent of this impact is heterogeneous across states, depending on their income levels. The consistency of the coefficients across one-step and two-step GMM estimators further enhances the credibility of the results.^12^ These results not only validate the economic significance of power reliability but also highlight the importance of targeted investment in grid infrastructure, especially in economically disadvantaged regions, to promote equitable and resilient growth.

### Power reliability and state-level income inequality

We now shift our attention to the relationship between power reliability and income inequality, as measured by the Gini Index, presented in Table 3. Since the Gini Index is used in levels while the power reliability measures are log-transformed, the estimated coefficients in this section are interpreted as semi-elasticities. The estimation results from both one-step and two-step System GMM methods provide consistent evidence that power interruptions are significantly associated with rising income inequality across U.S. states.

**Table 3:** Power reliability and state-level income inequality

Dependent variable: income inequality (Gini Index)						
Variable	SAIDI w/ MED	SAIFI w/ MED	CAIDI w/ MED	SAIDI w/o MED	SAIFI w/o MED	CAIDI w/o MED
*One-Step System GMM*						
Lagged Income Inequality (Gini Index)	− 0.012 (0.141)	0.250 (0.238)	− 0.021 (0.137)	0.052 (0.160)	0.305* (0.138)	0.100 (0.148)
Power Interruption	0.011** (0.004)	0.080* (0.044)	0.012* (0.006)	0.036 (0.027)	0.032* (0.017)	0.024 (0.016)
Unemployment Rate	0.002** (0.0006)	0.005* (0.002)	0.001* (0.006)	0.002* (0.001)	0.002*** (0.0005)	0.002*** (0.0007)
Electricity Price	0.002 (0.008)	0.056 (0.040)	0.002 (0.008)	0.010 (0.15)	0.003 (0.008)	0.029** (0.012)
Capital Outlay per Capita	0.010*** (0.002)	0.009*** (0.003)	0.010*** (0.002)	0.010*** (0.002)	0.008*** (0.002)	0.009*** (0.002)
AR(1) test	0.002	0.050	0.002	0.001	0.000	0.000
AR(2) test	0.379	0.176	0.458	0.191	0.072	0.236
Hansen J-test	0.582	0.942	0.263	0.301	0.081	0.271
Difference-in-Hansen test	0.693	0.472	0.625	0.174	0.213	0.142
Controls	Yes	Yes	Yes	Yes	Yes	Yes
Instruments	12	11	12	9	10	9
*Two-step system GMM*						
Lagged Income Inequality (Gini Index)	0.054 (0.162)	0.340 (0.214)	0.061 (0.163)	0.002 (0.172)	0.311 (0.227)	0.158 (0.128)
Power Interruption	0.014** (0.005)	0.092** (0.040)	0.016** (0.007)	0.060** (0.028)	0.025 (0.028)	0.018 (0.020)
Unemployment Rate	0.002*** (0.0006)	0.006** (0.002)	0.002*** (0.0007)	0.003** (0.001)	0.002** (0.0008)	0.002*** (0.0008)
Electricity Price	0.004 (0.009)	0.057 (0.039)	0.006 (0.009)	0.022 90.016)	0.026 (0.018)	0.028* (0.015)
Capital Outlay per Capita	0.009*** (0.002)	0.009*** (0.002)	0.008*** (0.002)	0.010*** (0.002)	0.006*** (0.002)	0.008*** (0.001)
AR (1) test	0.000	0.022	0.000	0.002	0.003	0.000
AR (2) test	0.274	0.122	0.260	0.085	0.195	0.303
Hansen J-test	0.582	0.942	0.263	0.301	0.084	0.271
Difference-in-Hansen test	0.693	0.783	0.625	0.174	0.477	0.142
Controls	Yes	Yes	Yes	Yes	Yes	Yes
Instruments	12	11		9	9	9
No. of Groups	50	50	50	50	50	50
Observations	450	450	450	450	450	450

^*^, ** and *** denote significance at the 10%-, 5%- and 1%-level. Each model regresses Income Inequality (Gini Index) on the log of lagged income inequality and the log of the power reliability variable by replacing each one while controlling for the log of the GDP in Eq. (1). Robust standard errors clustered at the state-level are in parentheses

Among the power reliability indicators, SAIFI (w/ MED) exhibits the strongest and most robust association with inequality. Specifically, a 1% increase in SAIFI (w/ MED) is associated with an increase in approximately 0.0008–0.0009 points in the Gini Index, corresponding to about a 0.17–0.20% increase relative to the sample mean. Power outages may disproportionately affect lower-income households through lost wages, limited ability to work remotely, lack of access to backup power sources, and greater exposure to service disruptions. In contrast, higher-income households and firms may be better able to smooth these shocks through savings, flexible work arrangements, and private mitigation technologies, such as generators or energy storage. As a result, frequent power interruptions amplify existing disparities and contribute to widening income inequality. This suggests that states experiencing more frequent outages tend to face greater income disparities, possibly due to the unequal capacity of households and communities to absorb the disruptions caused by frequent service interruptions.

CAIDI (w/ MED), which reflects the average time required to restore service, also shows a statistically significant and positive effect on income inequality, with estimated coefficients of 0.012 in the one-step model and 0.016 in the two-step model. Although these effects are smaller than those of SAIFI, they highlight the role of prolonged outages in worsening socioeconomic inequality. Likewise, SAIDI (w/ MED) yields modest but statistically significant coefficients of 0.011 (one-step) and 0.014 (two-step), indicating that both the frequency and the duration of power interruptions contribute to inequality outcomes.

When major event days are excluded, the magnitudes and statistical significance of the coefficients generally decline, suggesting that severe or widespread events are more consequential in exacerbating inequality than routine interruptions.

In summary, the results in Table 3 suggest that inadequate power reliability contributes to rising income inequality, particularly when service interruptions are frequent or prolonged. This relationship likely reflects the unequal ability of households to cope with power disruptions. Higher income households may have access to alternative energy sources, backup generators, or work from home flexibility, whereas lower-income households face more severe economic consequences. The consistency of these findings across multiple specifications highlights the importance of energy infrastructure not only as a technical asset but also as a determinant of social equity and economic inclusion.

Taken together, the analyses in Sects. 5.1 and 5.2 show that unreliable power supply restricts economic growth and contributes to increasing income disparities, particularly in economically lagging states. This highlights the importance of strengthening energy infrastructure and resilience, not only to boost productivity but also to support more equitable economic outcomes.

### Marginal effects

To further quantify the broader economic and distributional impacts of electricity supply disruptions in the U.S. economy, we employ a marginal effects analysis. Specifically, we examine the marginal effects of power reliability metrics—SAIDI, SAIFI, and CAIDI—on both state-level GDP and income inequality. The marginal effects on GDP capture the direct economic cost of power interruptions, estimating both short run and long-run output losses associated with increases in outage frequency and duration. The marginal effects on the Gini Index reveal how power reliability affects income distribution, with higher outage metrics linked to worsening inequality in most specifications. This approach provides a comprehensive view of the consequences of unreliable electricity supply, offering policy relevant estimates of both economic losses and equity implications.

We chose to use the two-step GMM approach over the one-step method for estimating the marginal effects of power interruptions on real GDP due to several key advantages. While one-step GMM provides consistent estimates, it assumes homoskedasticity and may be less efficient when this assumption is violated. The two-step GMM improves upon this by recalculating the weighting matrix with residuals from the first step, allowing for more efficient estimates even with heteroskedasticity. Additionally, two-step GMM provides robust standard errors, ensuring that test statistics and confidence intervals are more reliable. This method also reduces finite sample bias and enhances the reliability of diagnostic tests, such as the Hansen J-test.

#### Marginal effects of power interruption on state-level GDP

The marginal effects on GDP are summarized in Table 4. Among all power reliability indicators, SAIFI, which measures the frequency of power interruptions, has the most substantial and statistically significant negative impact on GDP. A one-unit increase in SAIFI (w/o MED) is associated with an estimated short-run GDP loss of $1,622.19 billion,^13^ which rises to $2,023.48 billion in the long run. These results suggest that frequent power interruptions disrupt economic activity in the short term and compound over time, resulting in substantial long-term economic consequences.

**Table 4:** Marginal effects of power interruption on state-level GDP

		Marginal effects on GDP			
	Two-step GMM coefficient (SE)	Short-run loss ($ B)	*P* value (SR)	Long-run loss ($ B)	*P* value ( LR)
	(1)	(2)	(3)	(4)	(5)
*With MED*					
SAIDI	− 0.079** (0.035)	30.82	0.025	752.84	0.863
SAIFI	− 3.517*** (0.449)	1375.03	0.000	1841.23	0.000
CAIDI	− 0.086** (0.041)	33.65	0.038	388.36	0.724
*Without MED*					
SAIDI	− 0.110** (0.044)	42.84	0.013	214.87	0.008
SAIFI	− 4.149*** (0.534)	1622.19	0.000	2023.48	0.000
CAIDI	− 0.150** (0.072)	58.53	0.038	317.75	0.073

^*^, **, and *** denote significance at the 10%-, 5%-, and 1%-levels. This table presents the marginal effects of power reliability on GDP in the U.S. from 2013 to 2022. SR refers to the short-run effect, and LR refers to the long-run effect

SAIDI, which reflects the total duration of interruptions, also shows a significant negative impact, albeit smaller in magnitude. A one-unit increase in SAIDI (w/o MED) corresponds to a short-run GDP loss of $42.84 billion and a long-run loss of $214.87 billion. CAIDI (w/o MED) is similarly associated with GDP losses of about $58.53 billion in the short run and $317.75 billion in the long run.

Overall, both the frequency and duration of outages negatively affect economic performance, but outage frequency (SAIFI) emerges as the most critical determinant. This may be because frequent outages have a more disruptive effect on business operations (e.g., due to repeated startup costs, etc.) thus reducing productivity and increasing financial losses (Ali et al. 2023). For example, firms subject to recurring outages may face lost output, operational inefficiencies, and revenue declines.^14^

#### Marginal effects of power interruption on state-level income inequality (Gini Index)

Table 5 reports the estimated marginal effects of power reliability indicators—SAIDI, CAIDI, and SAIFI—on income inequality, measured by the Gini Index.

**Table 5:** Marginal effects of power interruption on state-level Gini index

		Marginal Effects on Income Inequality			
	Two-Step GMM Coefficient (SE)	Short-run Effect	*P* value (SR)	Long-run Effect	*P* value (LR)
	(1)	(2)	(3)	(4)	(5)
*With MED*					
SAIDI	0.014** (0.005)	0.006	0.011	0.007	0.004
SAIFI	0.092** (0.040)	0.043	0.023	0.065	0.101
CAIDI	0.016** (0.007)	0.007	0.025	0.008	0.015
*Without MED*					
SAIDI	0.060** (0.028)	0.028	0.033	0.028	0.012
SAIFI	0.025 (0.028)	0.012	0.367	0.017	0.458
CAIDI	0.018 (0.020)	0.009	0.369	0.010	0.346

^*^, **, and *** denote significance at the 10%-, 5%-, and 1%-levels. This table presents the marginal effects of power reliability on Gini Index in the U.S. from 2013 to 2022. SR refers to the short-run effect, and LR refers to the long-run effect

Across all specifications, both short-run and long-run effects are positive, indicating that increases in power outages are associated with higher income inequality. Specifically, a 1 percent increase in the SAIDI (w/ MED) is associated with a short-run rise of 0.006 in the Gini Index (p = 0.011) and a long-run effect of 0.007 (p = 0.004), both statistically significant. When major events are excluded, these effects become stronger: 0.028 in both the short-run and long-run. Similar patterns emerge for CAIDI and SAIFI, but the effects without major event days are weaker and not statistically significant. Although the Gini Index is bounded and nonlinear, making marginal changes appear small in absolute terms, these shifts reflect meaningful changes in income distribution, especially in large populations.^15^

### Robustness checks and model validation using machine learning

To test the stability of our findings beyond the assumptions of System GMM, we conduct a sensitivity analysis using three machine learning (ML) methods: Ridge regression (Hastie, Tibshirani, & Friedman, 2009), XGBoost (Chen & Guestrin 2016), and Random Forest (Breiman 2001). These models are used for validation rather than causal inference, and provide flexible, data-driven benchmarks that can capture both linear and nonlinear relationships, handle multicollinearity, and identify the most influential predictors without relying on instrument validity or distributional assumptions. This allows us to assess whether power reliability remains a key predictor of state-level GDP and income inequality in nonparametric settings. Detailed variable importance rankings are presented in Appendices B1–B2, and performance metrics appear in Appendix C.

For the GDP models, Ridge regression (R^2^ = 0.9579, RMSE = 0.2149), XGBoost (R^2^ = 0.9550, RMSE = 0.2221), and Random Forest (R^2^ = 0.9555, RMSE = 0.2209) consistently rank log population, GDP per capita, and power reliability indicators—particularly SAIFI and CAIDI during major event days—among the most influential predictors. These results closely align with the System GMM estimates.^16^

For the Gini Index models, structural variables such as education and average wage emerge as leading predictors, with power reliability metrics playing a secondary but still meaningful role. Predictive performance is moderate—Ridge (R^2^ = 0.1533, RMSE = 0.0165), Random Forest (R^2^ = 0.2172, RMSE = 0.0157), and XGBoost (R^2^ = 0.0939, RMSE = 0.0168)—yet all three methods confirm that certain reliability indicators remain relevant in explaining inequality differences across states.

Overall, the machine learning validation confirms that the key relationships identified in our System GMM analysis are stable across a range of modeling approaches. While predictive accuracy is not the primary goal (Hastie, Tibshirani, & Friedman, 2009; Athey & Imbens 2019) the convergence of variable importance rankings—particularly for SAIFI, CAIDI, and core structural controls—enhances confidence in the robustness of our findings. These results suggest that improvements in power reliability remain economically significant in both parametric and nonparametric frameworks, reinforcing the policy relevance of targeted investments in grid infrastructure.

## Conclusion and policy implications

This study contributes to our understanding of the impact that electric power reliability has on state-level economic performance and income distribution across the U.S. Using a dynamic panel data framework and the System GMM estimator, we find robust evidence that increased frequency and duration of power interruptions are significantly associated with lower state-level GDP and higher income inequality. These findings are particularly salient given the increasing strain on electricity infrastructure due to extreme weather events and rising demand.

Our analysis indicates that a 1% increase in power interruptions, whether measured by the SAIDI, SAIFI, or CAIDI, results in an average GDP decline ranging from 0.07% to 3.7%. These estimates remain statistically significant in both short-run and long-run models, with frequency-based metrics (especially SAIFI w/o MED) having the largest economic effects. These effects are particularly pronounced in states with lower income, which appear less equipped to cope with service disruptions. Perhaps frequent interruptions create repetitive productivity losses, planning uncertainty, and operational inefficiencies that hinder both firms and households. By contrast, longer but less frequent outages may allow for more targeted mitigation and adaptation, leading to relatively less economic damage per unit of interruption.

The marginal effects are especially revealing. Specifically, a one-unit increase in SAIFI (w/o MED) is associated with a short-run GDP loss of roughly $1.62 trillion and a long-run loss of over $2.02 trillion. These substantial figures underscore the high economic cost of frequent outages, suggesting that power reliability is not merely a technical or engineering issue but a macroeconomic imperative.

The results also suggest that power unreliability worsens income inequality, with the Gini Index increasing by approximately 0.17% to 0.20% relative to the mean in response to a 1% increase in power interruptions. These findings are consistent with broader empirical evidence linking infrastructure quality to income distribution. For example, using cross-country panel data and dynamic estimation methods, Calderon and Chong (2004) find that both the quantity and quality of infrastructure, including transportation, energy, and telecommunications, are significantly associated with reductions in income inequality. Their results suggest that enhancing infrastructure access and performance can play a crucial role in promoting equitable development, particularly in contexts where disparities in service delivery exacerbate social inequalities. The observation of similar effects at the subnational level in the U.S. highlights the generalizability of this mechanism.

We also enhance the robustness of our findings using machine learning techniques, such as Ridge regression, XGBoost, and Random Forest. These data-driven models validate the predictive significance of power reliability indicators, even in flexible, nonlinear contexts. Across all three methods, power metrics like SAIFI and CAIDI consistently rank among the top predictors of both GDP and the Gini Index. From a policy perspective, these findings have direct implications for infrastructure investment, grid management, and resilience planning at the state level. Additionally, the results may assist in calculations of the value of lost load (VOLL) and provide useful information for benefit-cost analyses related to power transmission and distribution.

The implications are clear: Improvements in electricity reliability are not just about keeping the lights on, as they are crucial for sustaining economic growth. States that reduce the frequency and duration of outages may not only experience stronger economic performance but also limit the widening of income inequality. Policymakers should consider targeted investments in grid modernization, outage response systems, and preventive maintenance to alleviate both the economic and social costs of unreliable power.

Furthermore, our findings suggest that interventions should focus not only on extreme events (captured by Major Event Days) but also on routine, preventable outages. This distinction is important because routine outages are more amenable to policy intervention through maintenance, monitoring, and operational improvements than large-scale extreme events. Power interruptions occurring outside major events seem to have larger and more persistent economic effects. This highlights the importance of everyday resilience improvements—such as enhanced fault detection, decentralized energy storage, and automated grid controls—which can significantly reduce outage frequency and duration.

Overall, this study demonstrates that enhancing electricity reliability has multiple benefits: It contributes to macroeconomic stability and reduces structural inequality. Future work could expand this analysis by incorporating sector-specific vulnerabilities, employment effects, and the role of renewable energy integration; however, the current evidence already offers a compelling case for prioritizing power reliability in both economic and social policy agendas.

## Figures and Tables

**Fig. 3:**
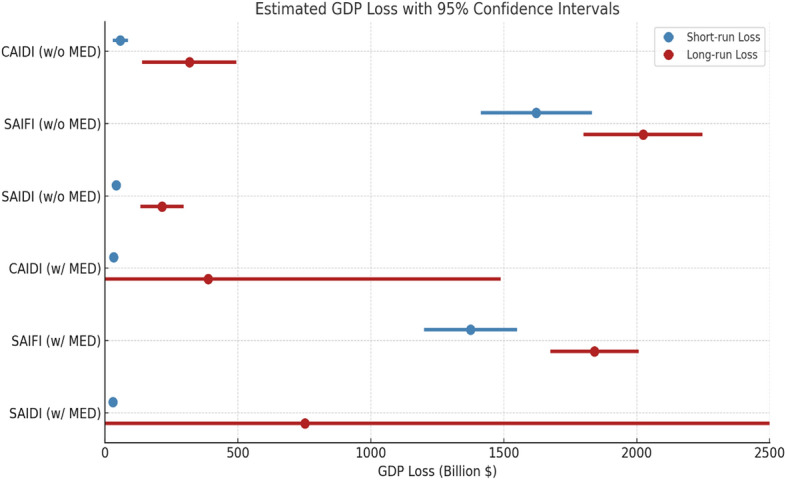
Estimated GDP loss with 95% confidence intervals

**Fig. 4:**
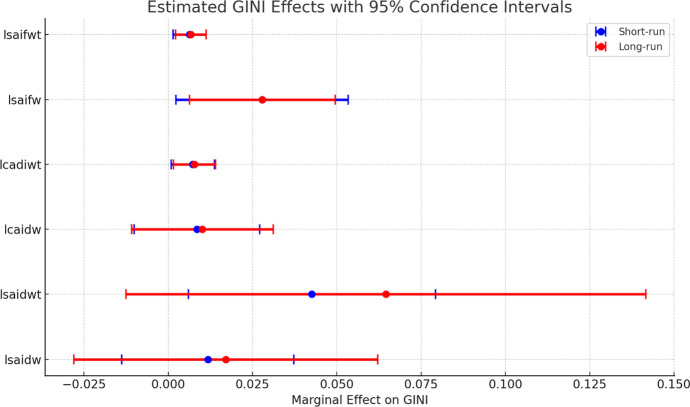
Estimated GINI effects with 95% confidence intervals

## Data Availability

The authors do not currently have permission to publicly share the data used in this analysis.

## Appendix

Figure 3, 4 and Table

**Table 6:** Variable descriptions, units, and data sources

Variable name	Justification/ proxy TYPE	Unit	Data source
Real gross domestic product (GDP)	Proxy for total economic activity and state-level output. Used to assess the macroeconomic consequences of power interruptions and infrastructure shocks	Thousands of chained (2017) dollars	BEA state GDP
Gini index	Measure of income inequality. Captures the distributional impact of economic factors, including unequal access to infrastructure, wages, and services	Unitless (0–1 scale)	ACS (U.S. census bureau)
GDP per capita	Economic output measure; captures the level of state economic performance	Thousands $ per person	BEA state GDP
SAIDI (w/ MED)	Infrastructure reliability; measures average outage duration (incl. major events)	Log minutes	U.S. DOE/EIA
SAIFI (w/ MED)	Infrastructure reliability; captures average number of interruptions per customer	Log interruptions	U.S. DOE/EIA
CAIDI (w/ MED)	Infrastructure efficiency; average restoration time, a proxy for grid resilience	Log minutes per interruption	U.S. DOE/EIA
SAIDI (w/o MED)	Infrastructure reliability (excluding major events); reveals baseline outage burden	Log minutes	U.S. DOE/EIA
SAIFI (w/o MED)	Baseline interruption frequency; useful for isolating routine reliability issues	Log Interruptions	U.S. DOE/EIA
CAIDI (w/o MED)	Baseline restoration time; reflects efficiency of power recovery operations	Log minutes per interruption	U.S. DOE/EIA
Unemployment rate	Labor market condition; higher rates may amplify vulnerability to outages	Percentage (%)	BLS (Local Area Unemployment Statistics)
Capital outlay per Capita	Public infrastructure investment; higher values may mitigate power disruption effects	Thousand $ per person	Census of governments
Urbanization rate	Urbanization; densely populated areas may experience different outage dynamics	Percentage (%)	ACS (U.S. census bureau)
Population	Population scale; reflects market size and service delivery scope	Log Total population	ACS / BEA
Electricity price	Electricity price; captures economic cost of energy and signals demand/supply issues	Cents per kWh	EIA (electric power monthly)
Labor force participation rate	Labor force participation; proxy for economic engagement and vulnerability	Percentage (%)	BLS
Manufacturing share	Industrial structure; manufacturing sectors are energy intensive and sensitive to reliability	Percentage of GSP	BEA state GDP by industry
Broadband Access Rate	Technological infrastructure; broadband is critical for economic resilience and digital access	Percentage (%)	ACS / FCC

ACS, American community survey; BEA, Bureau of economic analysis; BLS, Bureau of labor statistics; EIA, U.S. energy information administration; DOE, U.S. department of energy FCC, Federal communications commission; QCEW, Quarterly census of employment and wages; Census, U.S. census bureau; LAUS, Local area Unemployment statistics

6,

**Table 7:** Full set of descriptive statistics

Variable	Mean	Std. dev	Min	Max
SAIDI w/ MED	1.655	0.633	0	4.593
SAIDI w/o MED	1.101	0.311	0	2.309
CAIDI w/ MED	1.391	0.436	0	3.406
CAIDI w/o MED	1.034	0.178	0	1.753
SAIFI w/ MED	0.876	0.221	0	1.808
SAIFI w/o MED	0.742	0.182	0	1.482
Real GDP	12.325	1.048	10.358	14.968
Real GDP per capita	4.033	0.186	3.596	4.495
Gini index	0.465	0.019	0.41	0.52
Unemployment rate	4.896	1.738	1.99	13.72
Population	8.293	1.013	6.36	10.584
Urbanization rate	72.722	14.618	34.34	94.76
Electricity price	2.374	0.287	1.959	3.682
Labor Force participation rate	95.106	1.739	86.325	98.108
Manufacturing share	10.618	1.063	8.297	13.372
Capital outlay per capita	14.455	0.945	12.28	17.803
Broadband access rate	81.18	9.837	44.263	96.6

All values are presented in their logarithmic form, except the Gini Index and unemployment rate. Also note that 'w/' denotes 'with,' and 'w/o' denotes 'without,' as used in the table to indicate the inclusion or exclusion of specific factors. All power reliability measures are expressed in logged hours or logged interruptions, GDP variables are in logged real terms, and the Gini Index is used in levels for interpretability

7,

**Table 8:** Feature importance scores and coefficients for GDP prediction

Variable	Ridge coefficient	XGBoost importance	Random forest importance
Population	0.7426	0.9279	0.9802
Manufacturing share	0.1157	0.0143	0.0025
SAIFI w/	− 0.1091	0.0112	0.0004
SAIFI w/o	− 0.1445	0.0039	0.0005
SAIDI w/o	− 0.0153	0.0027	0.0014
SAIDI w/o * GDP per Capita	0.0658	0.0036	0.0023
CAIDI w/o * GDP per Capita	0.0361	0.0038	0.0021
Capital outlay per capita	0.0849	0.0091	0.0006
Urbanization rate	0.0779	0.0089	0.0059
Electricity price	0.0270	0.0069	0.0020
SAIFI w/ * GDP per Capita	0.0789	0.0004	0.0002
CAIDI w/ * GDP per Capita	0.0053	0.0022	0.0001
SAIFI w/o * GDP per Capita	0.0484	0.0013	0.0005
SAIDI w/ * GDP per capita	0.0010	0.0010	0.0001
Labor force participation rate	0.0063	0.0006	0.0001
Unemployment rate	− 0.0023	0.0005	0.0001
Share of broadband access	0.0045	0.0004	0.0001
CAIDI w/	0.0000	0.0000	0.0000

^8^,

**Table 9:** Feature importance scores and coefficients for Gini Index prediction

Variable	Ridge coefficient	XGBoost importance	Random forest importance
SAIDI w/	0.000800	0.008000	0.005800
SAIDI w/	− 0.000200	0.021800	0.034200
CAIDI w/	0.001000	0.006700	0.007800
CAIDI w/o	− 0.000900	0.022100	0.023200
SAIFI w/	0.000900	0.081600	0.037900
SAIFI w/o	0.001500	0.096800	0.040600
GDP per capita	− 0.001600	0.101600	0.164400
Unemployment rate	0.001800	0.018700	0.013200
Population	0.005600	0.304100	0.353400
Urbanization rate	0.000500	0.089300	0.078100
Electricity price	0.000500	0.091000	0.094500
Labor force participation rate	− 0.001700	0.027100	0.015600
Manufacturing share	0.000700	0.025600	0.020800
Capital outlay per capita	0.003800	0.030300	0.052400
Share of broadband access	− 0.001100	0.007100	0.011300
Education (bachelor’s or higher)	− 0.000500	0.016600	0.020400
State average wage	0.002500	0.051500	0.026200

^9^ and

**Table 10:** Machine learning model performance metrics (robustness check)

Model	R^2^ (CV Mean)—GDP	RMSE (CV)—GDP	R^2^ (CV Mean)—Gini	RMSE (CV)—Gini
Ridge Regression	0.9579	0.2149	0.1533	0.0165
XGBoost	0.9550	0.2221	0.0939	0.0168
Random Forest	0.9555	0.2209	0.2172	0.0157

CV = Cross-validation. Performance is strong and consistent across models for GDP. Predictive strength for Gini is modest, which may reflect indirect causal pathways or omitted latent variables not captured in the ML framework

10.

## Footnotes

1 On average, United States electricity customers experienced just over eight hours of electric power interruptions in 2022. When major event days (MED) are excluded, the average duration of interruptions customers experienced annually from 2013 to 2022 was consistently around two hours (U.S. Energy Information Administration: *Annual Electric Power Industry Report, 2023*).

2 Atems & Hotaling (2018) also find that power losses, whether from an inefficient electricity grid, poor electricity infrastructure, or theft, have a statistically significant negative relationship with economic growth. They suggest that encouraging more investment in the electricity transmission and distribution grid, developing microgrids in remote areas, and limiting electrity theft, may be beneficial policy strategies.

3 While power providers can use any method to calculate grid reliability, it is common to use the standards developed by the Institute of Electrical and Electronic Engineers (IEEE). Thus, we use the metrics that were calculated using the IEEE standards.

4 Non-momentary outages are those lasting more than five minutes, and minutes were converted into hours for appropriate system GMM analysis.

5 CAIDI is SAIDI divided by SAIFI.

6 It is considered a MED if: SAIDI > T_MED_ = e^(α+2.5β)^, where α is the log average of daily outages and β is the log standard deviation of five years data.

7 To improve model parsimony and address multicollinearity, several variables, such as the share of college-educated residents and state-level minimum wage, were excluded from the final specifications of the inequality regressions. These variables exhibited high variance inflation factors and were strongly correlated with retained covariates such as unemployment and average wages (Gujarati & Porter 2009). Their exclusion ensures coefficient stability and enhances the interpretability of the results.

8 Appendix A2 provides the full set of descriptive statistics for all the variables in the analyses.

9 We considered alternative estimators such as the Augmented Mean Group (AMG) approach. However, AMG is primarily designed for panels with longer time dimensions and focuses on addressing cross sectional dependence rather than endogeneity arising from lagged dependent variables. Given the relatively short time dimension of our panel (2013–2022) and the central role of endogeneity in the power reliability–growth–inequality nexus, System GMM provides a more appropriate identification framework.

10 We use robust standard errors, and the AR test confirms the expected first-order autocorrelation and no significant second-order autocorrelation, indicating that the instruments are valid and that the model specification is correct.

11 To enhance the robustness and precision of the inequality models, we excluded select covariates and interaction terms that exhibited high multicollinearity—identified through variance inflation factors and correlation diagnostics. In particular, the interaction between power interruptions and GDP per capita was removed from the GINI specification, as its inclusion led to weak instruments, inflated standard errors, and inconsistent estimates of key coefficients. This refinement improves model parsimony and ensures more reliable inference on the distributional consequences of power reliability.

12 While some model specifications contain a number of instruments (e.g., 52) that slightly exceeds the number of cross sectional units (50 states), the diagnostic results—including insignificant AR(2) tests and well-behaved Hansen J and Difference-in-Hansen statistics—support the validity of the instrument set. The small-sample adjusted two-step estimator of Windmeijer (2005) further enhances the reliability of standard errors. Although a high instrument count relative to the number of groups can, in some cases, lead to instrument proliferation and weaken the Hansen test (Roodman 2009), our results remain robust across specifications with different instrument counts and lag structures. Similar estimation practices can be found in the literature on energy and inclusive growth. For instance, (Medeiros & Ribeiro 2020) employ System GMM models with instrument counts exceeding the number of countries, yet report strong diagnostic performance and robust findings regarding the impact of energy access on inclusive growth at the global level.

13 For these calculations, we do not convert the variables into their logarithmic form.

14 We graphically present the estimated short-run and long-run GDP losses with 95% confidence intervals by reliability indicator (see Appendix D, Figure D1).

15 We visualize the distributional consequences of power unreliability in Appendix D, Figure D2, which illustrates how different types of outages affect income inequality in both the short and long run.

16 Control variables are consistent with those used in the main GMM specification. These include the unemployment rate, log of population, broadband access rate, labor force participation, manufacturing share, capital outlay per capita, electricity price, and urbanization rate.
